# Identification and validation of mitochondria-related genes in panvascular diseases

**DOI:** 10.3389/fmed.2025.1614342

**Published:** 2025-07-10

**Authors:** Yingfen Li, Shenzhou Ma, Guang Yang

**Affiliations:** ^1^Department of Gerontology, The First Affiliated Hospital of Shandong First Medical University and Shandong Provincial Qianfoshan Hospital, Jinan, China; ^2^Shandong First Medical University & Shandong Academy of Medical Sciences, Jinan, China

**Keywords:** panvascular diseases, peripheral artery disease, coronary artery disease, mitochondria-related genes, immune infiltration

## Abstract

Panvascular diseases represent a spectrum of vascular conditions where atherosclerosis plays a central role in the pathophysiology. This study focused on identifying differentially expressed genes (DEGs) related to mitochondria and key genes associated with peripheral artery disease (PAD) and coronary artery disease (CAD). This study identified MPV17 as a key mitochondrial gene bridging peripheral artery disease (PAD) and coronary artery disease (CAD). Analysis of GEO datasets revealed differentially expressed mitochondrial genes, with MPV17, FADD, HLCS, and PEX3 highlighted. A diagnostic nomogram, developed using LASSO and Random Forest methods, demonstrated high accuracy in predicting PAD and CAD (AUC >0.93). Furthermore, the study revealed significant alterations in immune cell infiltration associated with both diseases, suggesting a potential role for immune modulation in panvascular disease. MPV17 shows promise as a diagnostic marker for early identification and differentiation of these vascular conditions.

## 1 Introduction

Panvascular diseases include various vascular conditions that share a common underlying issue: atherosclerosis. This condition affects vital organs, including the heart, brain, kidneys, and limbs (1, 2). Recent advancements in medical specialization have resulted in the categorization of panvascular diseases—such as coronary artery disease (CAD), ischemic stroke, and peripheral artery disease (PAD)—into separate disciplines. This trend has often resulted in fragmented management strategies, which may hinder effective treatment and care for patients with interconnected conditions (3). This disciplinary separation tends to emphasize local lesions while neglecting systemic vascular changes, which can create significant disparities in diagnostic and therapeutic approaches (4). The concept of panvascular medicine promotes a holistic view of the body’s structure and function, aiming to understand the mechanisms underlying the emergence and progression of atherosclerotic diseases through the application of systems biology and multidimensional approaches (3, 5, 6). This paper focuses on the significance of adopting an integrated approach to PAD and CAD. It underscores the importance of acknowledging the interconnectedness of vascular health across various conditions and medical specialties.

PAD and CAD are two significant cardiovascular conditions that profoundly impact patients’ health and quality of life (7–9). These diseases can lead to severe symptoms, including limb pain, restricted mobility, and myocardial infarction, which can even endanger patients’ lives. As a result, they impose a substantial burden not only on individuals but also on society as a whole (10, 11). Current treatment options for PAD and CAD primarily consist of drug therapy, interventional therapies, and surgical procedures (12, 13). However, these approaches have inherent limitations regarding their effectiveness, potential side effects, and rates of disease recurrence. As a result, exploring innovative diagnostic and therapeutic strategies is essential for advancing healthcare (14, 15).

Previous research has highlighted the essential role of mitochondria in cellular energy metabolism (16), oxidative stress (17), and apoptosis (18), all of which are closely linked to the development of PAD and CAD (19). Alterations in the expression of genes associated with mitochondrial function may play a significant role in the development and progression of these conditions (20, 21). Consequently, this study aims to investigate mitochondrial-related genes to better understand their influence on PAD and CAD, offering a novel and promising area of research.

The aim of this study is to conduct a comprehensive analysis of datasets associated with PAD and CAD. This includes identifying differentially expressed genes (DEGs) associated with mitochondrial function and pinpointing key hub genes implicated in these diseases. Additionally, we will develop a diagnostic model that offers innovative approaches to improve the diagnosis and treatment of panvascular diseases.

## 2 Methods

### 2.1 Screening of differentially expressed genes

**Figure 1:**
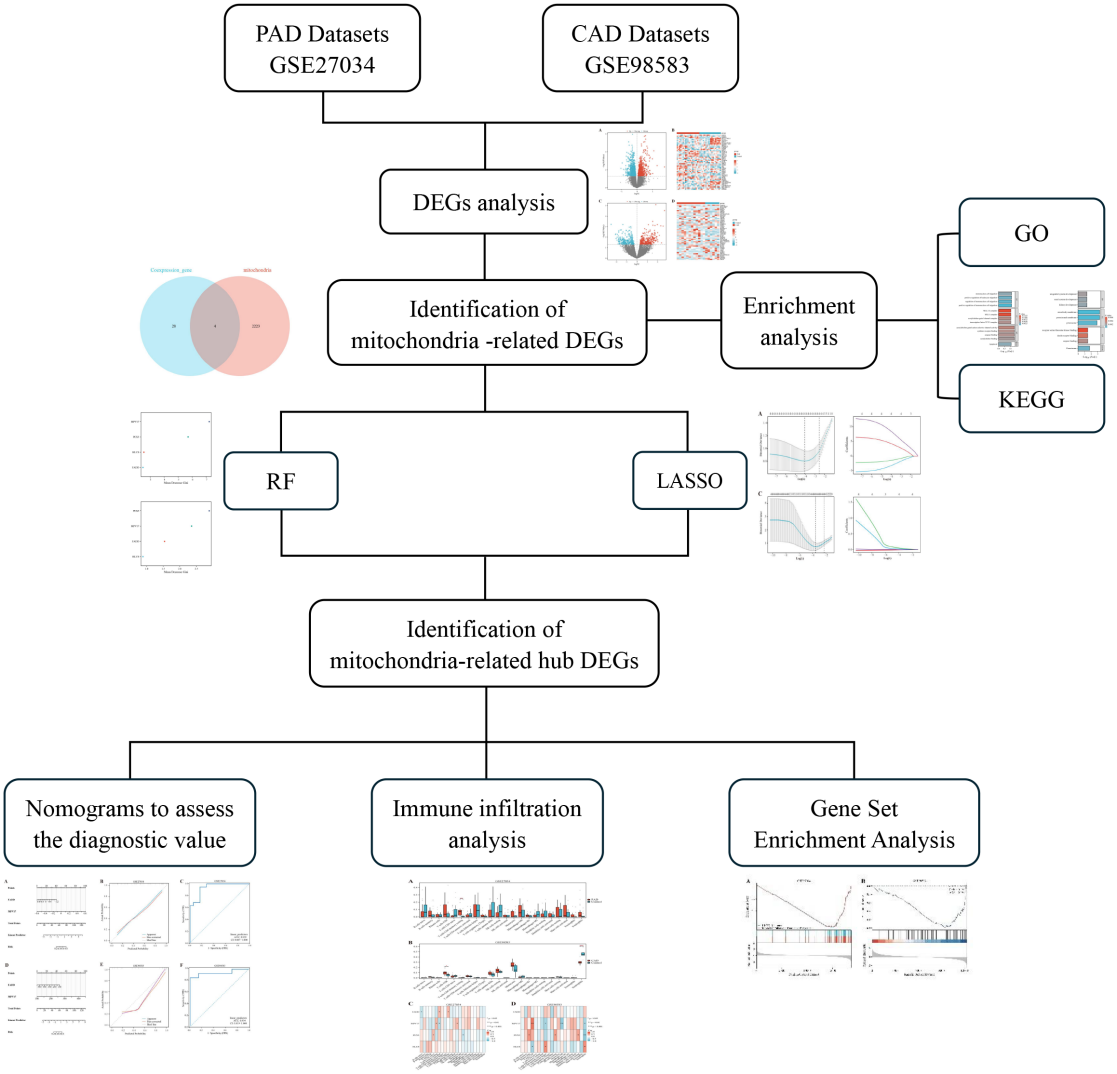
Flowchart of the study design.

We performed a search in the Gene Expression Omnibus (GEO) database (22)^1^ with the keywords “peripheral arterial disease” and “coronary artery disease” to identify relevant datasets for both PAD and CAD. To explore mitochondrial-related genes, we accessed the GeneCards database (23),^2^ which provides comprehensive information on genes, including their functions, pathways, and associated diseases. Our selection criteria restricted the datasets to those derived from human samples, with each dataset needing to contain a minimum of 10 samples. We specifically downloaded the GSE27034 (24) and GSE98583 (25) datasets, which correspond to the GPL570-55999 and GPL571-17391 platforms, respectively. The GSE27034 dataset comprises peripheral blood samples collected from 19 patients diagnosed with PAD and 18 healthy individuals serving as controls. In contrast, the GSE98583 dataset includes samples from 12 non-diabetic male patients with stable CAD and six samples from individuals with normal coronary angiogram. Figure 1 presents an overview of the study’s methodology.

The analysis of differentially expressed genes (DEGs) from the GSE27034 and GSE98583 datasets was conducted using the “limma” package in R software. For both datasets, the criteria for screening DEGs were set to a |log2FC| >0 and adjusted to *p* < 0.05 (26). Following the analysis, visualizations in the form of volcano plots and heat maps were generated using the R packages “ggplot2” and “ComplexHeatmap” (27). These visualizations help to illustrate the expression profiles of the DEGs and allow for an easier comparison between the affected and control groups.

### 2.2 Screening and identification of mitochondria-related DEGs

We identified 2,227 genes associated with mitochondria by querying the GeneCard database and using a relevance score threshold of greater than 1 as our screening criterion. Subsequently, we conducted a screening for DEGs that displayed consistent expression patterns across the datasets. To identify the mitochondria-related DEGs, we intersected the list of co-expressed DEGs with the 2,227 mitochondria-related genes we obtained from the GeneCard database.

### 2.3 Enrichment analysis of DEGs

We performed functional enrichment analyses using Gene Ontology (GO) and the Kyoto Encyclopedia of Genes and Genomes (KEGG) on the selected differential genes, employing the ClusterProfiler package in R. The screening criterion was established with a significance threshold of *p* < 0.05 (28–30).

### 2.4 Selection and identification of mitochondria-related hub DEGs

To effectively select and evaluate key genes associated with prognosis, we utilized the least absolute shrinkage and selection operator (LASSO) method, implemented through the “glmnet” package in R. This approach involved determining the optimal penalty coefficients using 10-fold cross-validation to construct robust regression models. Additionally, we developed random forest (RF) models with the “randomForest” package in R to enhance our analysis and confirm the prognostic significance of the identified genes.

### 2.5 Nomogram development for diagnostic models of PAD and CAD

The diagnostic value of mitochondria-associated hub DEGs in PAD and CAD datasets was assessed using a nomogram constructed with the “rms” package in R. This nomogram allows for the visualization of the relationship between the DEGs and the likelihood of disease occurrence. In order to evaluate the reliability of this diagnostic model, we employed receiver operating characteristic (ROC) curves to measure the model’s sensitivity and specificity, as well as calibration curves to assess how well the predicted probabilities align with the actual outcomes.

### 2.6 Evaluation of immune infiltration in PAD and CAD

The CIBERSORT program was employed to assess the levels of immune infiltration across 22 distinct immune cell types in patients with PAD and CAD (31). Additionally, heat maps were utilized to illustrate the relationship between the mitochondria-related hub DEGs and the 22 immune cell types. To further understand the potential biological pathways and processes linked to these hub genes, we performed gene enrichment analysis (32).

## 3 Results

### 3.1 Screening of DEGs

**Figure 2:**
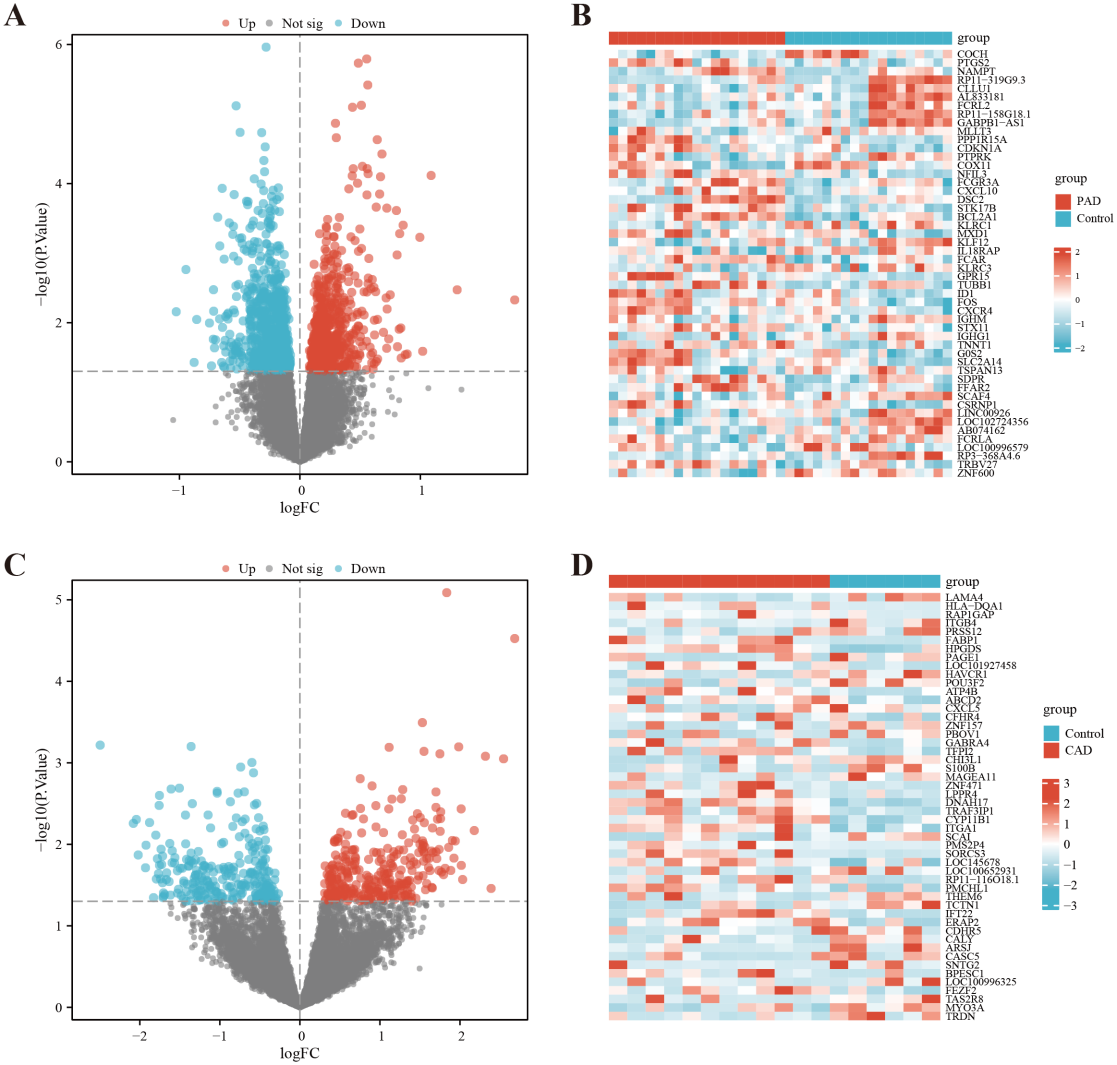
Identification of differentially expressed genes (DEGs). DEG heatmaps and volcano plots for the **(A,B)** PAD and **(C,D)** CAD datasets.

**Figure 3:**
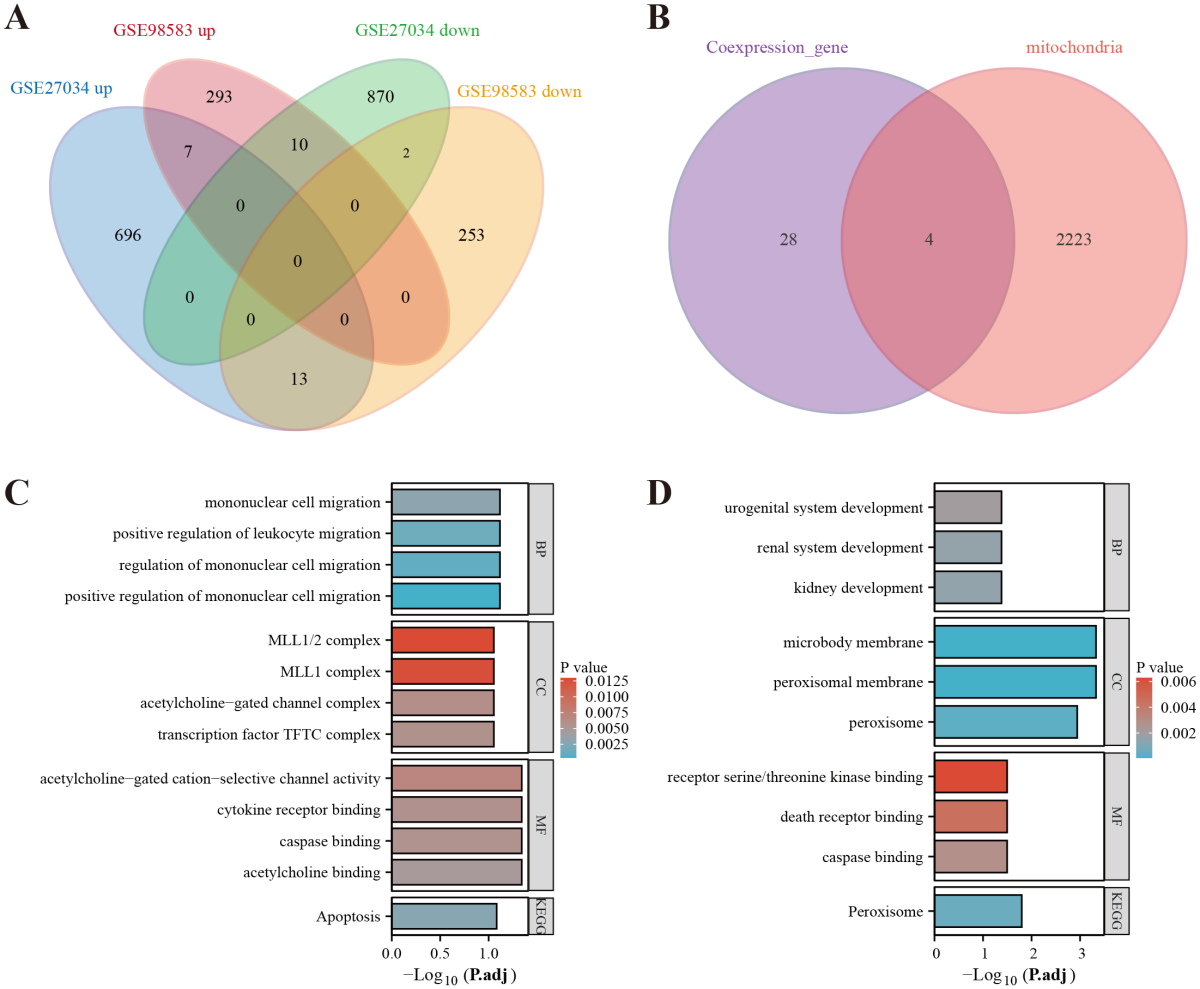
Identification of mitochondria-related DEGs and functional enrichment between PAD and CAD. **(A)** Venn diagram of the DEGs. **(B)** Venn diagram of the mitochondria-related DEGs. **(C)** GO and KEGG enrichment analyses of DEGs with the same expression trends. **(D)** GO and KEGG enrichment analyses of the mitochondria-related DEGs.

By analyzing the GSE27034 dataset, a total of 1,598 DEGs were identified, including 716 up-regulated genes and 882 down-regulated genes. In contrast, the GSE98583 dataset showed 578 DEGs, with 310 upregulated genes and 268 downregulated genes. To visually represent the expression profiles of the DEGs from both datasets, we created volcano plots, where upregulated genes are highlighted in red and downregulated genes in blue. Additionally, we utilized heat maps to illustrate the top 50 differentially expressed genes from both GSE27034 and GSE98583, allowing for a comparative overview of the gene expression patterns associated with these datasets (see Figure 2). To further illustrate the overlap between the identified DEGs, Venn diagrams are presented in Figure 3A.

### 3.2 Screening and identification of mitochondria-related DEGs

As a result of screening the genes associated with PAD and CAD, we identified DEGs that exhibited similar expression trends. These DEGs were interleaved with genes related to mitochondrial function, as presented in Figure 3B. We identified four mitochondria-related DEGs: MPV17, FADD, HLCS, and PEX3. DEGs with a consistent expression trend in PAD and CAD were significantly enriched in multiple biological processes (BP), primarily in pathways related to mononuclear cell migration, positive regulation of leukocyte migration, and regulation of mononuclear cell migration. In terms of cellular components (CC), these DEGs were enriched in complexes such as the MLL1/2 complex, MLL1 complex, acetylcholine-gated channel complex, and the transcription factor TFTC complex. Additionally, within molecular function (MF) analysis, the genes were associated with acetylcholine-gated cation-selective channel activity, cytokine receptor binding, caspase binding, and acetylcholine binding. The KEGG enrichment analysis demonstrated a significant enrichment of these genes in the apoptosis pathway, as illustrated in Figure 3C. Conversely, the mitochondria-related DEGs showed a predominant enrichment for biological processes associated with kidney development, urogenital system development, and renal system development in the biological process (BP) enrichment analysis. For cellular components, these genes were related to microbody membrane, peroxisomal membrane, and peroxisome. Their molecular functions encompassed receptor serine/threonine kinase binding, death receptor binding, and caspase binding. The KEGG pathway analysis for these genes revealed significant enrichment in the Peroxisome pathway, depicted in Figure 3D.

### 3.3 Identifying mitochondrial-related hub genes and assessing their diagnostic values

**Figure 4:**
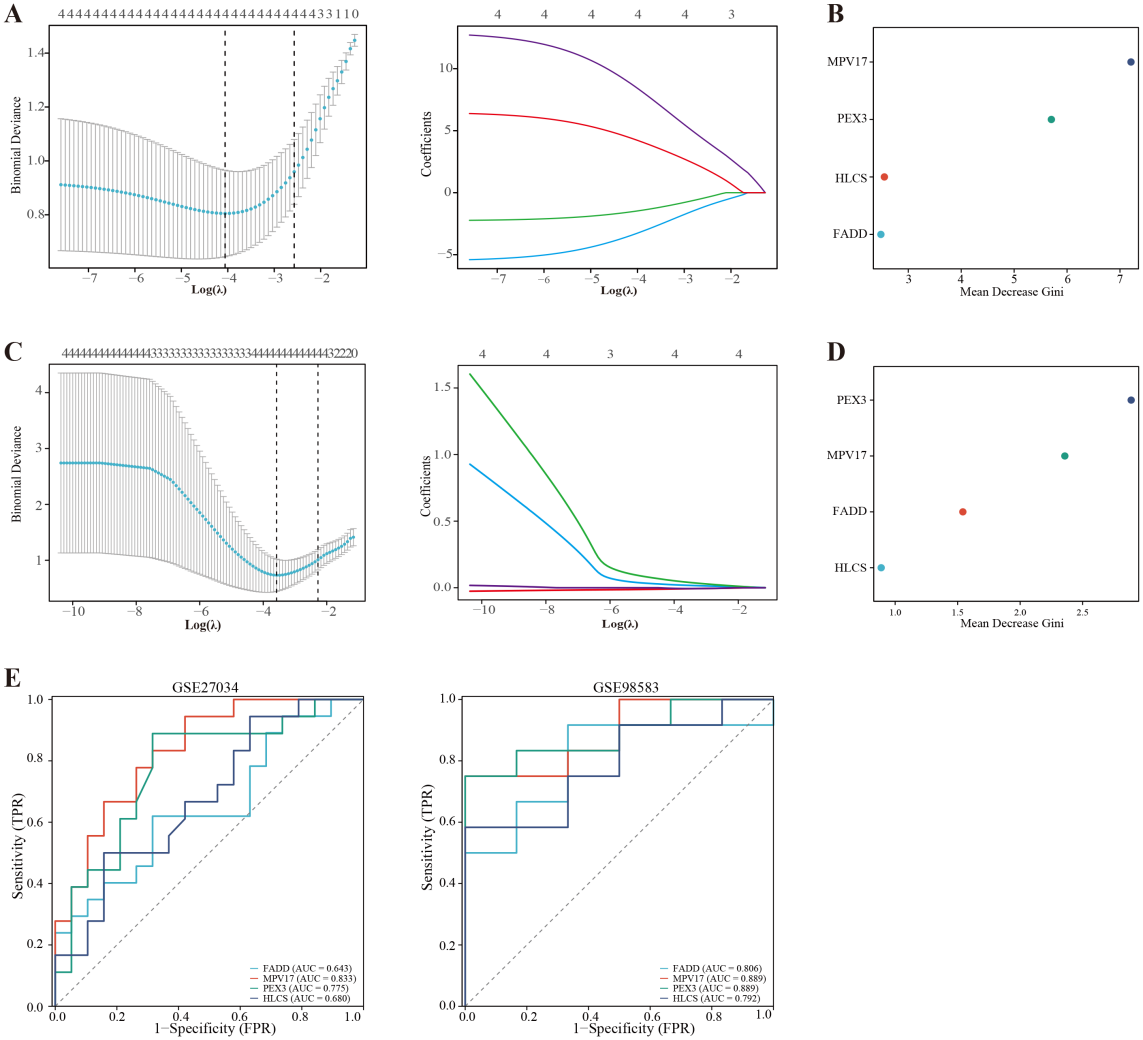
Screening of mitochondria-related hub genes and evaluation of their diagnostic values. **(A)** LASSO analysis for screening mitochondria-related hub genes in GSE27034. **(B)** Identification of mitochondria-related hub genes according to the importance of variables by random forest (RF) analysis of GSE27034. **(C)** LASSO analysis for screening mitochondria-related hub genes in GSE98583. **(D)** Identification of mitochondria-related hub genes according to the importance of variables by RF analysis of GSE98583. **(E)** Receiver operating characteristic (ROC) curves of the four hub genes to assess their diagnostic values in the GSE27034 and GSE98583 datasets.

**Figure 5:**
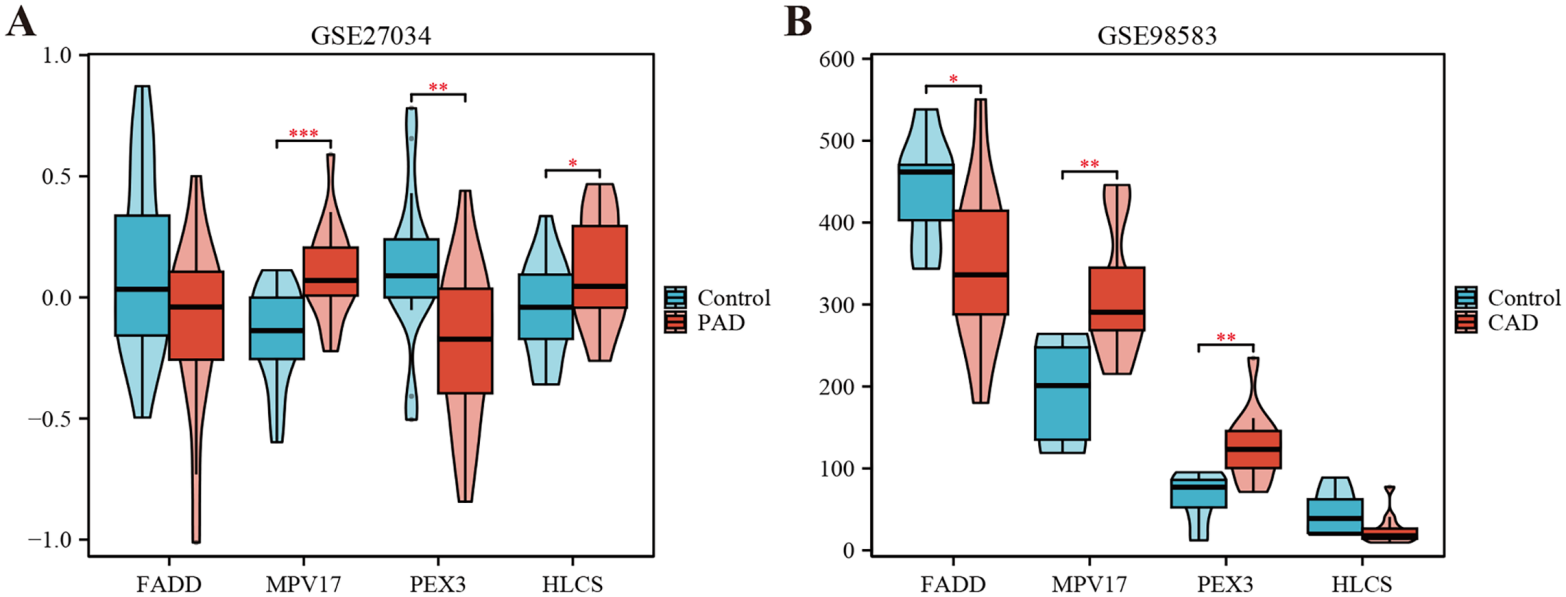
Expression levels of the four hub genes in the GSE27034 and GSE98583 datasets. **(A)** Expression level of FADD, MPV17, PEX3 and HLCS in GSE27034. **(B)** Expression level of FADD, MPV17, PEX3 and HLCS in GSE98583. ^*^p < 0.05, ^**^p < 0.01, and ^***^p < 0.001.

Using the LASSO regression analysis and the RF method, we screened 4 mitochondria-related genes associated with PAD and CAD: MPV17, FADD, HLCS, and PEX3 (as shown in Figures 4A–D). To assess the diagnostic potential of these four hub genes, we employed Receiver Operating Characteristic (ROC) curve analysis, the results of which are illustrated in Figure 4E. Among these genes, MPV17 was found to be significantly upregulated in the context of PAD and CAD (refer to Figure 5). In contrast, FADD and HLCS did not show a significant difference in expression levels between PAD patients and healthy individuals. Furthermore, the expression levels of PEX3 exhibited inconsistencies between the two groups.

### 3.4 Nomogram development for diagnostic models of PAD and CAD

**Figure 6:**
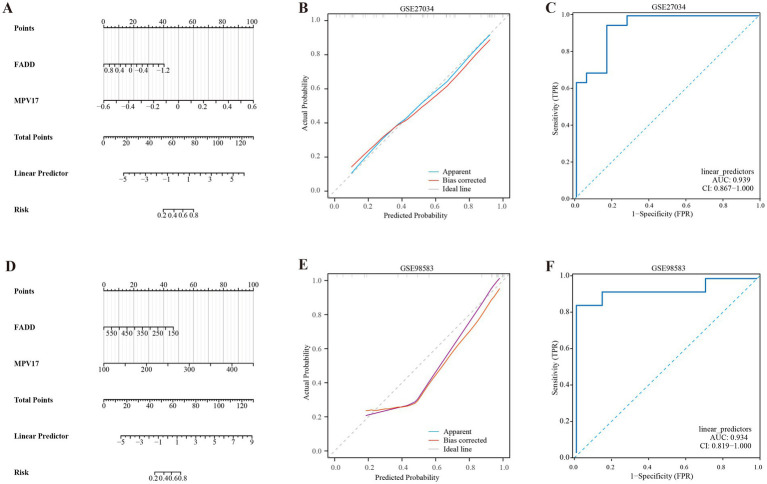
Development of the diagnostic nomogram model. **(A)** Nomogram predicting the probability of PAD. **(B)** Calibration curves of the PAD risk models. **(C)** ROC curve of the PAD risk model. **(D)** Nomogram predicting the probability of CAD. **(E)** Calibration curves of the CAD risk models. **(F)** ROC curve of the CAD risk model.

The nomogram model was developed by integrating the points of the hub genes of PAD and CAD, as illustrated in Figures 6A,D. The bias-corrected calibration curves demonstrated a strong alignment with the ideal calibration curve, signifying that our model exhibits excellent calibration (shown in Figures 6B,E). Additionally, the model’s performance was further validated by evaluating the area under the curve (AUC) for the GSE27034 and GSE98583 datasets, which yielded values of 0.939 and 0.934, respectively, as depicted in Figures 6C,F, indicating that the model demonstrates high reliability.

### 3.5 Evaluation of immune infiltrations in PAD and CAD

**Figure 7:**
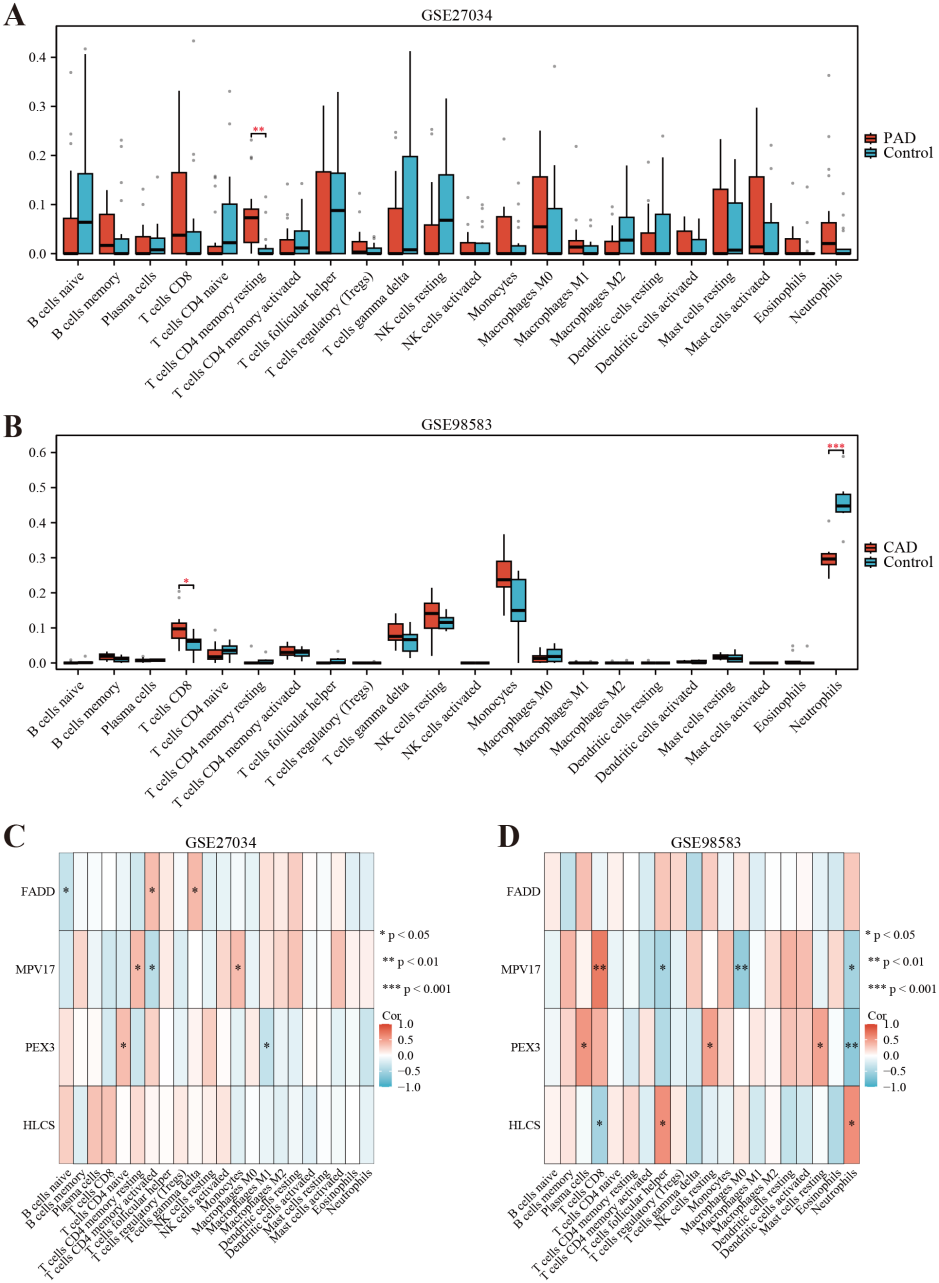
Immune cell infiltration analyses in PAD and CAD. **(A)** Boxplot showing the comparison of 22 kinds of immune cells between PAD and the control group. **(B)** Boxplot showing the comparison of 22 kinds of immune cells between CAD and the control group. **(C)** Heatmap representing the associations of the differentially infiltrated immune cells with the four hub genes in PAD for the threshold of *p* < 0.05, ^*^p < 0.05, ^**^p < 0.01, and ^***^p < 0.001. **(D)** Heatmap representing the associations of the differentially infiltrated immune cells with the four hub genes in CAD for the threshold of *p* < 0.05, ^*^p < 0.05, ^**^p < 0.01, and ^***^p < 0.001.

**Figure 8:**
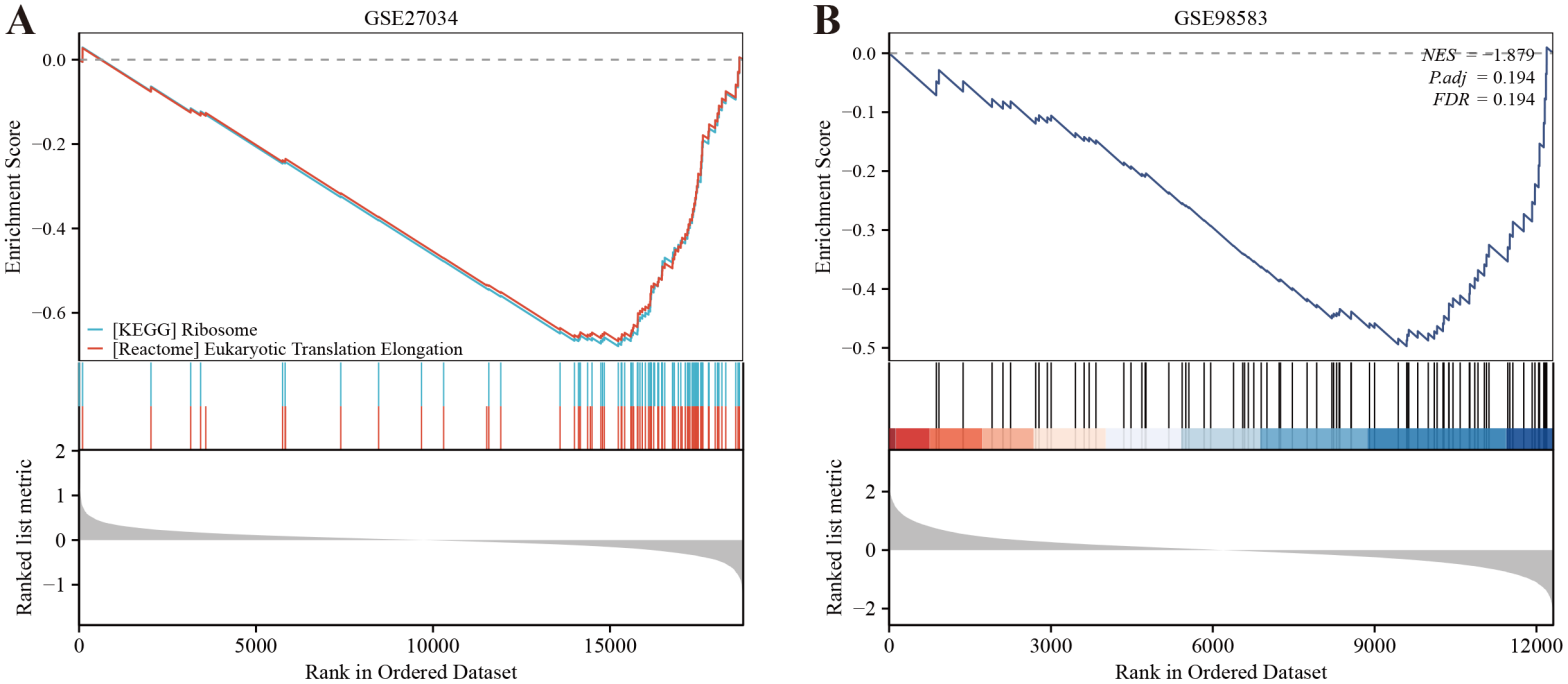
Gene set enrichment analysis (GSEA) for MPV17 in **(A)** PAD and **(B)** CAD.

We employed the CIBERSORT deconvolution algorithm to evaluate immune cell infiltrations in cases compared to controls within the GSE27034 and GSE98583 datasets. The analysis revealed that the PAD group exhibited significantly higher resting memory CD4^+^ T cell numbers (Figure 7A provides a visual representation of this). In contrast, patients with CAD demonstrated a significant elevation in CD8^+^ T cells (illustrated in Figure 7B). Additionally, our analysis indicated that the identified hub gene, MPV17, had strong associations with multiple immune cell types (depicted in Figures 7C,D). Further, gene enrichment analysis highlighted that MPV17 was predominantly enriched in pathways related to the ribosome and eukaryotic translation elongation in the context of PAD (as seen in Figure 8A). Conversely, in patients with CAD, MPV17 showed enrichment in the REG GR pathway (shown in Figure 8B).

## 4 Discussion

Panvascular medicine is an emerging discipline that emphasizes the multidisciplinary integration of knowledge and practices concerning atherosclerotic diseases (3). Over the years, it has transitioned from a theoretical framework into clinical practice, highlighting the need for an all-encompassing management model that encompasses prevention, diagnosis, treatment, and prognosis of vascular diseases (1, 5). PAD and CAD are two significant cardiovascular conditions that significantly affect patients’ health and overall quality of life (33, 34). This study focused on identifying the mitochondria-related hub genes of PAD and CAD. Understanding these hub genes may facilitate the development of targeted therapies and enhance diagnostic accuracy, ultimately improving patient outcomes.

The focus on mitochondria-related genes in the context of PAD and CAD is vital, as mitochondrial dysfunction has become widely acknowledged as a significant factor in the development of diverse cardiovascular conditions. Research has shown that impaired mitochondrial function can lead to a host of detrimental effects, including reduced energy production (35), heightened oxidative stress, and altered calcium homeostasis (36). These factors can exacerbate endothelial dysfunction, promote inflammation, and accelerate atherogenesis, thus playing a crucial role in the onset and advancement of vascular diseases (37). This study aims to enhance our understanding of the underlying mechanisms of these diseases by examining specific genes, and identify potential diagnostic markers and therapeutic targets.

This study presents a detailed investigation into the molecular mechanisms associated with PAD and CAD. Utilizing datasets from the GEO and mitochondria-related genes obtained from GeneCard. Through differential gene expression analysis, a significant number of DEGs were identified in the GSE27034 and GSE98583 datasets. The results presented as heat maps and volcano plots facilitated the visualization and interpretation of these alterations. Several potential biomarkers were highlighted, including MPV17, FADD, HLCS, and PEX3, which are of considerable interest in the context of PAD and CAD. Notably, only MPV17 demonstrated a consistent expression trend across both diseases when analyzing the data. MPV17 is a protein located in the inner membrane of mitochondria. A deficiency of this protein can result in a reduction of mitochondrial DNA (mtDNA) and an increase in the levels of reactive oxygen species (ROS) (38), which contributes to atherosclerosis (39). Existing literature supports MPV17’s essential function in maintaining mitochondrial genome stability (40) and regulating redox homeostasis (41), both critical factors in atherosclerotic development. Furthermore, the development of a nomogram based on these key mitochondrial-related differential genes shows promise as a diagnostic tool for PAD and CAD. The high AUC values (0.939 and 0.934 for the two datasets) indicate good diagnostic performance.

The functional enrichment analysis of DEGs using GO and KEGG provided valuable insights into their biological roles and associated pathways (42). Notably, the apoptosis pathway emerged as a significant area of enrichment. This pathway is essential for determining cell fate, and disruptions in apoptotic processes may significantly exacerbate of PAD and CAD (43). Another important pathway identified in the analysis is the peroxisome pathway, which is involved in multiple metabolic processes. Research suggests that dysfunction in peroxisomes contributes to atherosclerosis through various mechanisms. For instance, a decreased ability to perform β-oxidation can result in the accumulation of very-long-chain fatty acids (VLCFAs) (44), which activates the TLR4/NF-κB pathway and initiates inflammatory responses (45). Furthermore, disruptions in hydrogen peroxide metabolism can activate the NLRP3 inflammasome (46), working synergistically with mitochondrial reactive oxygen species to promote endothelial apoptosis (47). In addition, the pathways related to urogenital system development, renal system development, and kidney development—identified in the biological process analysis of mitochondria-related DEGs—underscore the potential involvement of these systems in the pathogenesis of PAD and CAD. These results suggest that disturbances in these developmental processes may contribute to the onset and progression of both diseases.

We performed a deconvolution analysis using the CIBERSORT tool to assess the immune infiltration of 22 immune cells. Firstly, the observed significant increase in resting memory CD4^+^ T cells within the peripheral artery disease (PAD) group is noteworthy. These cells are a specific subset of T lymphocytes vital for coordinating immune responses. Their elevated presence may indicate an enhanced immune vigilance in individuals with PAD, suggesting potential adaptations in the immune system to address the underlying disease processes (48). In the context of PAD, the increased presence of these cells may suggest an ongoing immune response within the affected tissues. This could potentially be related to the pathophysiological processes underlying PAD, such as inflammation and tissue damage. Secondly, the significant elevation of CD8^+^ T cells in CAD patients is another important finding. CD8^+^ T cells, commonly referred to as cytotoxic T lymphocytes, are crucial for the direct elimination of target cells (49). Similar to previous findings, CD8^+^ T cells were significantly elevated in CAD (50). Research indicates that CD8^+^ T cells contribute to atherosclerosis through various mechanisms: while the secretion of IFN-γ activates pro-inflammatory macrophage phenotypes, which can accelerate necrotic core formation (51). Furthermore, single-cell RNA sequencing has shown that CD8^+^ T cells promote the dedifferentiation of smooth muscle cells (SMCs), driving them toward macrophage-like and osteoblast-like phenotypes that favor calcification (52). Clinical data demonstrate that an elevated frequency of CD8^+^ CD57^+^ T cells in the peripheral blood of patients who have experienced acute myocardial infarction (MI) and its correlation with 6-month cardiovascular mortality emphasizes the significance of these cells in CAD (53). Finally, the gene set enrichment analysis revealed that MPV17 is enriched in ribosome and eukaryotic translation elongation in PAD, while it is enriched in the REG GR pathway in CAD patients. These findings suggest that MPV17 may have distinct functional roles in the pathogenesis of PAD and CAD.

This study has several limitations that need to be recognized. First, the absence of wet experiments raises concerns regarding the reliability and reproducibility of the results. Second, the limited sample size may hinder the generalizability of the findings. While the chosen datasets offer valuable information for analysis, the limited sample count may weaken the strength of the findings. Moreover, the selection of the dataset may contain inherent biases, which highlights the need for further validation with larger and more diverse samples. Additionally, the focus on known mitochondrial-related genes may lead to the exclusion of other significant genes. Finally, the model’s establishment and validation relied primarily on statistical methods, without clinical validation to support its effectiveness.

## 5 Conclusion

This study significantly advances our understanding of the pathophysiological mechanisms of panvascular disease. It offers innovative ideas and potential biomarkers for its clinical diagnosis and treatment. We identified MPV17 as a mitochondrial-related hub gene between CAD and PAD. Its high diagnostic value indicates its potential as a biomarker for the early detection and differentiation of these vascular disorders. Future research should integrate wet experiments with multi-omics data to more comprehensively investigate the pathogenesis of panvascular disease, thereby developing more accurate and effective diagnostic and therapeutic approaches.

## Data Availability

Publicly available datasets were analyzed in this study. This data can be found at: https://www.ncbi.nlm.nih.gov/geo/query/acc.cgi?acc=GSE27034; https://www.ncbi.nlm.nih.gov/geo/query/acc.cgi?acc=GSE98583.

## Footnotes

^1^ http://www.ncbi.nlm.nih.gov/geo

^2^ https://www.genecards.org/
